# Fasting Plasma Glucose and Its Relationship to Anthropometric Phenotype in Women Diagnosed with Gestational Diabetes According to IADPSG Criteria

**DOI:** 10.3390/life13010137

**Published:** 2023-01-04

**Authors:** Ondrej Krystynik, Dominika Macakova, Lubica Cibickova, David Karasek

**Affiliations:** Third Department of Internal Medicine—Nephrology, Rheumatology and Endocrinology, University Hospital Olomouc and Faculty of Medicine and Dentistry, Palacký University Olomouc, 77900 Olomouc, Czech Republic

**Keywords:** gestational diabetes, first-trimester fasting glucose, OGTT, gestational weight gain, perinatal outcomes

## Abstract

Background: Gestational diabetes mellitus (GDM) is characterized by new-onset hyperglycemia in pregnancy. According to the International Association of Diabetes and Pregnancy Study Groups (IADPSG) recommendations, GDM may be diagnosed based on repeatedly increased fasting glucose levels in the first trimester, or later, the detection of increased fasting glucose and/or increased glucose levels during a 75 g oral glucose tolerance test (OGTT). The study aimed to assess whether differences may be found between women diagnosed with GDM by fasting glucose or glucose challenge tests in early or late pregnancy. Methods: The retrospective observational study enrolled 418 women diagnosed with GDM in accordance with the IADPSG criteria: early pregnancy fasting plasma glucose (FPG) ≥ 5.1 mmol/L; late pregnancy FPG ≥ 5.1 mmol/L (0 min) and/or postprandial plasma glucose (PPG) ≥ 10.0 mmol/L (60 min), PPG ≥ 8.5 mmol/L (120 min) 75 g OGTT. The analyses included anthropometric parameters at the beginning and during pregnancy, laboratory values of glycated hemoglobin, fructosamine, birth weight measures and the presence of neonatal complications. Results: There were significant differences in body weight (78.3 ± 19.1; 74.0 ± 16.7; 67.2 ± 15.7 kg) and body mass index (BMI) (27.9 ± 6.6; 26.4 ± 5.8; 24.4 ± 5.2 kg/m^2^) in early pregnancy. Differences were also found in gestational weight gain (9.3 ± 6.8 vs. 12.4 ± 6.9 vs. 11.1 ± 4.7 kg) and the need for insulin therapy (14.7%; 7.1%; 4.0%). The study revealed no difference in the presence of neonatal complications but differences in birth weight (3372.2 ± 552.2 vs. 3415.6 ± 529.0 vs. 3199.0 ± 560.5 g). Conclusions: Gestational diabetes, characterized by FPG ≥ 5.1 mmol/L in early pregnancy, is associated with higher body weight and BMI at the beginning of pregnancy as well as with a higher risk for insulin therapy and increased birth weight.

## 1. Introduction

Gestational diabetes (GDM) belongs to the group of endocrine disorders characterized by new-onset hyperglycemia first detected during pregnancy [1]. The pathophysiology of GDM is a complex and still not a fully understood issue. However, it appears that an increase in insulin resistance and an inability of beta cells to respond with sufficient insulin secretion are crucial factors for development of GDM in predisposed pregnant women [2]. As insulin resistance physiologically increases in the second half of pregnancy, GDM is most likely to be detected during this period. The condition is associated with a higher risk of pregnancy complications for both mothers and fetuses. It has been shown to increase the risk of metabolic disorders, especially type 2 diabetes mellitus, obesity and arterial hypertension and cardiovascular complications in the later lives of mothers and their babies [3,4,5].

Screening for and diagnosing GDM have changed considerably over the years. It should be noted that an international consensus has still not been reached in this area. According to recommendations on the diagnosis and classification of hyperglycemia in pregnancy published in 2010 by the International Association of Diabetes and Pregnancy Study Groups (IADPSG), GDM screening should be performed at 24–28 weeks of gestation. For the screening, a 75 g oral glucose tolerance test (OGTT) is recommended. Each diagnostic threshold is associated with a risk of complications at an odds ratio of 1.75, as shown by the HAPO study [3]. Besides the OGTT performed at the specified time of pregnancy, however, fasting glucose levels should be measured as soon after conception as possible, that is in the first trimester. The purpose is to identify apparent, previously unrecognized diabetes and to treat it early. At the same time, however, pregnant women with mild fasting hyperglycemia may also be identified, corresponding to diagnostic thresholds revealed by an OGTT in the second half of pregnancy. Based on these recommendations, these women are monitored in the same way as those diagnosed with GDM later in their pregnancy, although not enough studies have been performed to show the benefit of treating GDM early in pregnancy [6].

Using the same diagnostic thresholds for fasting glucose in early pregnancy and in its second half remains rather controversial. This is mainly because unlike the clearly higher risks of complications from elevated fasting glucose in the second half pregnancy, the association has not been confirmed in early gestation. Moreover, it is apparent that the early diagnosis of GDM made with fasting glucose tests in the first trimester has only limited correspondence to positive diagnostic results of a two-hour OGTT subsequently performed in the second half of pregnancy [7,8]. At the same time, the different approaches to diagnosing GDM are not considered at all during further monitoring or therapy. Moreover, it is unclear whether early and intensive treatment of GDM may contribute to the risk of fetal growth restriction and low weight gain in pregnant women [9].

The present study aimed to assess whether different ways of diagnosing GDM (Groups: early FPG, late FPG and late PPG) are able to identify women with different anthropometric phenotypes at the time of diagnosis. Another objective was to determine whether different ways of diagnosing GDM in these women are associated with differences in the course of pregnancy and the incidence of neonatal complications.

## 2. Materials and Methods

### 2.1. Study Design, Inclusion and Exclusion Criteria

A retrospective observational study was designed in accordance with the 2008 revision of the Declaration of Helsinki. University Hospital Olomouc medical records were searched to obtain information on women monitored for impaired carbohydrate metabolism during their pregnancy. Based on national recommendations, all pregnant women in the Czech Republic are screened for GDM regardless of the presence of risk factors for its development. The study enrolled only women diagnosed with GDM using the IADPSG recommendations who could be classified into three groups by the test method (Table 1).

We excluded women diagnosed with diabetes prior to their pregnancy (type 1 or 2 diabetes mellitus, maturity-onset diabetes of the young) as well as those meeting the criteria of apparent diabetes in the current pregnancy (FPG ≥ 7.0 mmol/L and/or post-OGTT glucose levels ≥ 11.1 mmol/L).

During their first visit, all the women were educated on basic dietary and lifestyle measures. Insulin therapy was only initiated if at least one of the following criteria was met: (1) FPG > 5.3 mmol/L; one-hour PPG repeatedly 7.6 mmol/L or two-hour PPG repeatedly >6.6 mmol/L. None of the enrolled patients received oral antidiabetic drugs. The review of medical records yielded information on the patients’ history (age, parity) and risk factors for the development of diabetes (family history of diabetes mellitus, history of GDM, birth weight > 4000 g). Early pregnancy anthropometric data (weight, height, BMI) were obtained from records on the first prenatal visit. Information on weight gains and GDM management (diet, insulin therapy) came from subsequent antenatal appointments. Data on birth weight, type of delivery (vaginal or cesarean), neonatal complications (neonatal hypoglycemia, infant jaundice) were retrieved from gynecology department records.

### 2.2. Laboratory Methods

Biochemical parameters (glucose, glycated hemoglobin and fructosamine levels) were analyzed using the Cobas 8000 system (Roche, Mannheim, Germany). Samples for the analyses were collected in the morning following at least 12-h fasting during the first prenatal visit.

### 2.3. Statistical Methods

Continuous variables are expressed as the mean, median and standard deviation. Differences in continuous variables among the groups were analyzed using the Kruskal–Wallis test. Differences in discrete variables among the groups were assessed by the chi-squared test on contingency tables. Spearman’s coefficient was used to express the correlation rate. All hypotheses were tested at a 0.05 level of significance. Data were assessed using MATLAB Version 7.5.0.342 (R2007b).

## 3. Results

### 3.1. Classification by GDM Diagnostic Method

**Figure 1:**
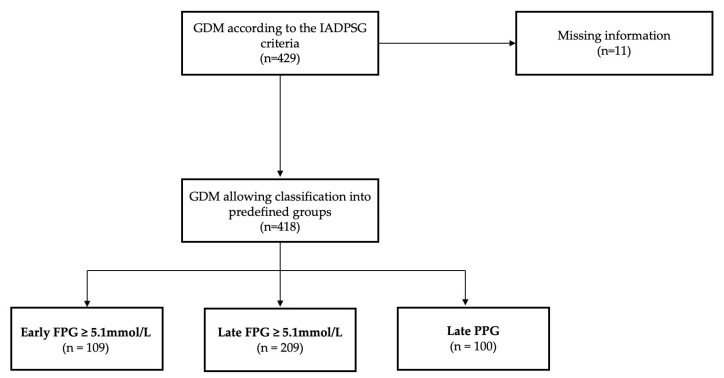
The sample classified into groups by GDM diagnostic method.

In the sample, GDM was predominantly diagnosed by increased FPG levels (early FPG and late FPG groups), namely in 73.9% of cases. At the same time, more than a quarter of the women (26.1%) were identified in early pregnancy (Figure 1).

### 3.2. Basis Sample Characteristics

The statistical analysis first focused on comparing all three groups in early pregnancy. The results are shown in Table 2. The women in the groups did not differ in terms of age, parity, presence of risk factors for the development of diabetes or metabolic control parameters (glycated hemoglobin levels) at the time of GDM diagnosis. However, there were differences in weight (i.e., BMI) at the beginning of their pregnancy. For women in all groups, early pregnancy BMI was positively correlated with glycated hemoglobin levels at the time of GDM diagnosis (ρ = 0.36, *p* < 0.001; ρ = 0.23, *p* < 0.001; ρ = 0.30, *p* < 0.001).

### 3.3. Course of Pregnancy

There were significant differences in overall weight gain among the groups of women with GDM. Moreover, in those diagnosed by increased FPG levels (early FPG and late FPG groups), this weight gain was negatively associated with BMI at the beginning of pregnancy (ρ = −0.25, *p* < 0.05; ρ = −0.21, *p* < 0.001). Similarly, there were significant differences in birth weight among the groups. In the early FPG and late FPG groups (GDM diagnosed based on increased FPG levels), birth weight was positively correlated with early pregnancy BMI (ρ = 0.27, *p* < 0.05; ρ = 0.23, *p* < 0.001). Insulin therapy was more frequently used to control GDM in women with increased fasting glucose levels (early FPG and late FPG groups) and was associated with body weight (ρ = 0.19, *p* < 0.001), BMI (ρ = 0.24, *p* < 0.001) and glycated hemoglobin at the time of GDM diagnosis (ρ = 0.30, *p* < 0.001). In the present study, different ways of diagnosing GDM had no effect on the incidence of the neonatal complications studied. Thus, the incidence was comparable in the three groups. The results are shown in Table 3.

## 4. Discussion

Using the IADPSG criteria for GDM in early pregnancy, we can identify a group of pregnant women who show differences in anthropometric parameters and course of pregnancy compared with women who were diagnosed later in pregnancy. In our study, we showed that early GDM diagnosis (FPG ≥ 5.1 mmol/L) is associated with increased body weight and BMI at the beginning of pregnancy. At the same time, early GDM diagnosis is accompanied by more frequent insulin treatment and higher birth weight. There was no association between the method of GDM diagnosis and the incidence of neonatal complications.

The main period of pregnancy in which the screening and diagnosis of GDM is carried out is the period between the 24th and 28th week of pregnancy. There is indisputable scientific evidence for the association between hyperglycemia during this period of pregnancy and late maternal and neonatal complications. At the same time, there is a known association between hyperglycemia and the incidence of congenital malformations in women with T1DM in early pregnancy. Therefore, there is concern about the negative impact of mild hyperglycemia in early pregnancy on fetal development and the subsequent course of pregnancy. Despite efforts to reach consensus, the way of diagnosing GDM in early pregnancy remains a rather controversial topic. Neither has a suitable screening test been found, nor have diagnostic thresholds been agreed upon for glucose levels in early gestation that would be clearly associated with a higher risk for complications in both mothers and fetuses later in the pregnancy [9]. Currently, screening for glucose metabolism impairment in early pregnancy is mainly focused on the detection and adequate treatment of overt diabetes. If overt diabetes is ruled out during the first antenatal visit, it is recommended to focus on pregnant women with FPG above 6.1 mmol/L because of the increased risk of developing GDM later in pregnancy and the increased risk of maternal and neonatal complications [10]. With regard to the cause of GDM, three different phenotypes are currently considered: prevailing insulin resistance, impaired insulin secretion or their combination. These changes have been observed in both early and late pregnancy. An important finding is that women with dominant impaired insulin sensitivity are at a higher risk for GDM-related pregnancy complications [2,11]. However, it is yet unknown whether this proven pathophysiological difference exists in response to standard GDM therapy.

As seen from the study results, the clinical use of the IADPSG criteria for diagnosing GDM facilitated identification of three groups of pregnant women that differed in anthropometric parameters at the time of GDM diagnosis as well as in how their pregnancy progressed. The strength of the study mainly lies in including pregnant women with an early GDM diagnosis made through universal screening in the first trimester (early FPG group), that is not only a population of pregnant women at a higher risk for developing diabetes. This is how 25% of women in the sample were identified. However, information on the global prevalence of hyperglycemia diagnosed this way in early gestation is missing, mainly due to persisting differences in GDM diagnostic methods. Among overweight women (BMI ≥ 29), 22.9% were diagnosed with early GDM using the IADPSG criteria. In the sample, FPG levels of 5.1 mmol/L or more contributed to the diagnosis in 78.5% of cases [12].

In the present study, increased FPG levels identified women with higher body weight and BMI at the beginning of their pregnancy when mothers-to-be were screened irrespective of presence or absence of GDM risk factors (i.e., including overweight or obesity). This finding is consistent with results of previous studies showing an association between GDM diagnosed by increased FPG levels and mean BMI levels suggestive of an overweight condition [2,7,12]. Thus, pre-pregnancy body weight and BMI seem to be a strong predictor for increased FPG detected in early gestation. Maternal obesity appears to be associated with increased insulin secretion (C-peptide levels) and decreased insulin sensitivity [13]. However, it appears that obesity in early pregnancy may also influence adipokines production. In our previous study we demonstrated increased adipocyte fatty acid-binding protein and decreased adiponectin levels and its correlation with visceral adiposity in women with impaired FPG in early pregnancy [14]. In contrast to higher body weight in early pregnancy, the overall weight gain was lowest in women with early GDM diagnosis in the present study. This may be explained by longer dietary restrictions as a form of GDM management. Our study did not include a control group of pregnant women with GDM who did not undergo nutritional or pharmacological intervention. Thus, the assessment of treatment effect is very limited. There is also a clear negative correlation between overall weight gain and BMI in early pregnancy. In women with early GDM diagnosis, however, insulin pharmacotherapy was initiated more frequently. This is consistent with earlier observations [15]. This may be explained by a limited effect of recommended dietary carbohydrate restriction on fasting glucose levels that repeatedly exceeded the recommended values and were a reason for therapy initiation. However, the initiation of pharmacological treatment is based on the physician’s decision. Therefore, the influence of selection bias on the outcome cannot be completely excluded.

Early GDM diagnosis was not associated with increased glycated hemoglobin levels. In the present study, the mean glycated hemoglobin level at diagnosis was within the range typical for normal glucose metabolism. It is, therefore, unlikely that using the IADPSG criteria in early pregnancy would have identified women with impaired glucose metabolism prior to their gestation. However, there is a clear positive correlation of glycated hemoglobin levels with body weight and BMI at the beginning of pregnancy. In contrast, there were significant differences in fructosamine levels. This may be explained by a drop in albumin concentrations in the second half of pregnancy (i.e., at the time of GDM diagnosis in the late FPG and late FPG groups) (14). Since fructosamine levels predominantly reflect albumin glycation, the differences in fructosamine concentrations are more likely to be associated with changes in albumin concentrations rather than the actual increase in non-enzymatic glycation occurring 2–3 weeks prior to sample collection. Given the retrospective design of the study, however, fructosamine measurements could not be corrected for albumin concentrations at the time of GDM diagnosis.

There were no statistically significant differences in the incidence of selected perinatal complications among the groups in our study. This is in contrast to results from a meta-analysis of 13 studies showing an increased relative risk of perinatal mortality and neonatal hypoglycemia in women with early GDM diagnosis compared with those diagnosed with GDM in the second half of their pregnancy [15]. The difference may be explained by the overall time of monitoring and treatment of women with GDM. However, it was not possible to assess the severity of GDM, total insulin dose or changes in glycemic profile as these data could not be obtained in a retrospective study. Early GDM diagnosis was also associated with higher birth weight. This may be due to previously described fetal hyperinsulinemia resulting from insulin resistance and more frequent insulin therapy [2,16]. Macrosomia (birth weight > 4000 g) was not associated with any of the groups defined in the present study. On the other hand, it was correlated with BMI levels, age and glycated hemoglobin levels at the time of GDM diagnosis. Due to the retrospective design of our study, it was not possible to perform OGTT between 24 and 28 weeks of gestation in women who were diagnosed with GDM early in pregnancy based on fasting glycaemia (early FPG group). As suggested by some studies, there is limited correlation between first trimester fasting glycaemia and 2-h 75 g OGTT in the diagnosis of GDM. Therefore, the presence of women in the early FPG group who would not meet the diagnostic criteria for GDM in the second half of pregnancy cannot be excluded and may have influenced the incidence of the observed outcomes.

## 5. Conclusions

Gestational diabetes, characterized by increased fasting plasma glucose levels (≥5.1 mmol/L) in early pregnancy according to the IADPSG criteria, is associated with increased body weight and BMI at the beginning of pregnancy as well as with a higher risk for insulin therapy and increased birth weight. The present study failed to show its impact on the likelihood of neonatal complications or fetal macrosomia.

## Figures and Tables

**Table 1:** The sample classified into groups by GDM diagnostic method.

Group	Gestational Age	Test Method	Glucose Level
Early FPG	1–13 weeks	fasting glucose	FPG ≥ 5.1 mmol/L
Late FPG	24–28 weeks	OGTT–minute 0	FPG ≥ 5.1 mmol/L
Late PPG	24–28 weeks	OGTT–minute 60 OGTT–minute 120	PPG ≥ 10.0 mmol/L PPG ≥ 8.5 mmol/L

OGTT = oral glucose tolerance test; FPG = fasting plasma glucose; PPG = postprandial plasma glucose.

**Table 2:** Basic sample characteristics. Anthropometric and laboratory analyses at diagnosis.

	Entire Sample	Early FPG ≥ 5.1 mmol/L	Late FPG ≥ 5.1 mmol/L	Late PPG	*p*-Value
Age	31.6 (32) ± 5.32	31.1 (31) ± 5.1	31.8 (32) ± 5.5	31.4 (31) ± 4.9	ns
Family history of DM (%)	59.2 (180/304)	36 (26/72)	44 (66/150)	40 (29/73)	ns
Weight (kg)	73.8 (70.8) ± 17.7	78.3 (76.0) ± 19.1	74.2 (72) ± 16.7	67.2 (62) ± 15.7	<0.001
BMI (kg/m^2^)	26.3 (25) ± 6.0	27.9 (27.2) ± 6.6	26.4 (25.0) ± 5.8	24.4 (22.8) ± 5.2	<0.001
HbA1c (mmol/mol)	31.1 (31) ± 4.0	31.8 (31) ± 4.8	30.7 (31) ± 3.8	30.8 (31) ± 3.2	ns
Fructosamine (μmol/L)	200.4 (199) ± 17.7	211.7 (211) ± 20.3	195.3 (194) ± 15.4	197.3 (197) ± 13.7	<0.001

DM = diabetes mellitus; BMI = body mass index; HbA1c = glycated hemoglobin. Data shown as the mean (median) ± standard deviation. Numbers of patients in groups: early FPG (*n* = 109), late FPG (*n* = 209), late PPG (*n* = 100). Differences in discrete variables among the groups (early FPG vs. late FPG vs. late PPG) were assessed by the chi-squared test on contingency tables; *p* < 0.05 is considered to be statistically significant.

**Table 3:** Body weight changes, need for treatment and neonatal complications.

	Early FPG ≥ 5.1 mmol/L	Late FPG ≥ 5.1 mmol/L	Late PPG	*p*-Value
Weight gain (kg)	9.32 (9.0) ± 6.76	12.73 (11.0) ± 8.47	11.05 (10.0) ± 4.66	<0.05
Insulin therapy (%)	14.7 (16/109)	7.2 (15/209)	4 (4/100)	<0.05
Birth weight (g)	3372.2 ± 552.2	3415.6 ± 529.0	3199.0 ± 560.5	<0.05
Emergency cesarean (%)	12.5 (9/72)	19.9 (30/151)	18.9 (14/74)	ns
Neonatal hypoglycemia (%)	14.1 (10/71)	10 (15/150)	7 (5/71)	ns
Neonatal jaundice (%)	43.1 (31/72)	41.1 (62/151)	47.3 (35/74)	ns

Data shown as the mean (median) ± standard deviation or as percentages (*n*/*N*). Numbers of patients in groups: Early FPG (*n* = 109), Late FPG (*n* = 209), Late PPG (*n* = 100). Differences in discrete variables among the groups (Early FPG vs. Late FPG vs. Late PPG) were assessed by the chi-squared test on contingency tables; *p* < 0.05 is considered to be statistically significant.

## Data Availability

The data used in this manuscript are available upon request.
